# Behavioral Epigenetics: Perspectives Based on Experience-Dependent Epigenetic Inheritance

**DOI:** 10.3390/epigenomes3030018

**Published:** 2019-08-23

**Authors:** You-Yuan Pang, Rita Jui-Hsien Lu, Pao-Yang Chen

**Affiliations:** 1 Institute of Plant and Microbial Biology, Academia Sinica, Taipei 11529, Taiwan; 2 Department of Agronomy, National Taiwan University, Taipei 10617, Taiwan

**Keywords:** behavioral epigenetics, epigenetic inheritance, experience-dependent epigenetics, epigenetic transgenerational inheritance, germline, somatic cells

## Abstract

Epigenetic regulation plays an important role in gene regulation, and epigenetic markers such as DNA methylation and histone modifications are generally described as switches that regulate gene expression. Behavioral epigenetics is defined as the study of how epigenetic alterations induced by experience and environmental stress may affect animal behavior. It studies epigenetic alterations due to environmental enrichment. Generally, molecular processes underlying epigenetic regulation in behavioral epigenetics include DNA methylation, post-translational histone modifications, noncoding RNA activity, and other unknown molecular processes. Whether the inheritance of epigenetic features will occur is a crucial question. In general, the mechanism underlying inheritance can be explained by two main phenomena: Germline-mediated epigenetic inheritance and interact epigenetic inheritance of somatic cells through germline. In this review, we focus on examining behavioral epigenetics based on its possible modes of inheritance and discuss the considerations in the research of epigenetic transgenerational inheritance.

## 1. Background—Behavioral Epigenetics

Epigenetics is the term that is widely used to describe the DNA-related molecular processes that may alter gene expression without changing the DNA sequence. In general, epigenetic modifications can be inherited both mitotically and meiotically. These epigenetic modifications play essential roles in gene regulation in which case epigenetic marks such as DNA methylation and histone modifications are often described as switches that regulate gene expression [1]. Aberrant changes in the epigenetic profile might induce abnormal gene silencing. Subsequently, physiological regulation, such as cellular differentiation, genomic imprinting, genome stability, and even behavior can be potentially affected. As studies have shown, similar physiological regulatory mechanisms are also observed in the offspring of affected organisms, proving that aberrant changes in the epigenetic profile are heritable.

In fact, several comprehensive studies have noted that environmental factors are closely associated with epigenetic regulations. Diet [2], toxicants [3], pollutants [4], and irradiation [5] are such factors that trigger alterations in epigenetic profiles. Interestingly, environment enrichments such as adverse experiences have also been proposed as factors that may affect the epigenetic profile [6]. For instance, childhood abuse and maternal separation are linked to aberrant DNA methylation profiles in the hypothalamic-pituitary-adrenal (HPA) stress response axis [7,8]. HPA axis is a complex set of direct influences and feedback interactions among the hypothalamus, pituitary gland, and adrenal glands, and it plays an important role in the reaction to various kind of stress. As a result, aberrant epigenetic regulation can disrupt the HPA axis activity, leading to an increased risk of suicide [9]. This type of epigenetic regulation induced by experience or enriched environment has attracted the attention of many epigenetic researchers.

To address this interesting phenomenon, researchers have recently worked on the concept of “behavioral epigenetics”. Behavioral epigenetics is defined as the study of how epigenetic alterations induced by experience and environmental stress may affect animal behavior, which describes the epigenetic alterations caused by environmental enrichments [5]. Environmental enrichment generally relates to the provision of environmental stimuli that promotes the expression of species-appropriate behavior and mental activities [10]. Furthermore, there is accumulating evidence of the presence of metastable epialleles in both plants and animals, which have been proven to be able to affect the activity of adjacent alleles [11,12,13]. Furthermore, some of these metastable epialleles are found to be associated with parent-of-origin effects and transgenesis in epigenetic inheritance [14]. However, the underlying mechanism of the transmission of epigenetic traits remains poorly understood. Therefore, herein, we study behavioral epigenetics by broadly surveying the research on epigenetic regulation induced by enriched environment and identifying the possible epigenetic marks involved. Moreover, the potentially transmissible pathways underlying behavioral epigenetics are highlighted in this review as well. We also suggest considerations in the establishment of a transgenerational epigenetic inheritance model in order to narrow the outlook for future behavioral epigenetic studies.

## 2. Epigenetic Regulation Triggered by Environmental Enrichments

Generally, the molecular processes that potentially underlie epigenetic regulation in behavioral epigenetics include DNA methylation, post-translational histone modifications, noncoding RNA, and other as yet unknown molecular processes. In addition, prion proteins [15] and microbiota [16] have also been reported to be involved in epigenetic regulation, despite the relative impact of these factors on behavioral epigenetics still remaining open to speculation. Over the last few years, several studies on environmentally-mediated epigenetic regulation have expanded the knowledge regarding behavioral epigenetics (see Table 1 for a summary of several reported cases of behavioral epigenetics). Herein, we describe the alterations in epigenetic markers induced by various environmental enrichments.

### 2.1. DNA Methylation

In mammalian epigenetic studies, the earliest known epigenetic marker that still remains is considered to be DNA methylation. DNA methylation is a process in which a methyl group from S-adenosyl-L-methionine (SAM) attaches to DNA nucleotide bases. DNA methylation sites occur predominantly on cytosine residues of CpG dinucleotides, which are unevenly distributed across the mammalian genome. Interestingly, clusters of unmethylated CpG sites in CpG-rich regions feature CpG islands (CGIs), which usually overlap with promoters. In a comprehensive study, the methylation markers that occur around gene promoter regions were seen to be frequently linked to gene-silencing events [25]. The DNA methylation profile has always been an important focus in epigenetic studies because the methylation state can further influence genome regulation [26].

In the field of behavioral epigenetics, exposure to adverse experiences during the postpartum period is often associated with aberrant methylation profiles of the genes encoding the glucocorticoid receptor (GCR) in both human and mouse models [7,8]. Studies on a human model have suggested that males exposed to adverse experiences during childhood exhibit hypermethylation of the GCR gene *NR3C1* [7]. In this study, hypermethylation of the *NR3C1* gene was seen to co-occur with the decreasing expression of the GC receptor 1F variant. The hypermethylated state of *NR3C1* disrupted the regulation of the transcription factor NGFI-A, a crucial response element of the GC receptor in the regulation of the postnatal cortisol stress response. Adverse maternal care provided during childhood increased the methylation of *NR3C1* gene and decreased the binding of NGFI-A to the promoter of the *NR3C1* gene. While the lack of methylation on the *NR3C1* gene cancels the effect of maternal care on NGFI-A binding status, the transcriptional activation of *NR3C1* gene is normally regulated as a result. The manipulations of methylation status suggest that differences in maternal care result in different methylation profiles and further affect the gene expression. Similarly, in a mouse model, NGFI-A was inhibited when the DNA methylation state was altered by variations in maternal care during the postpartum period [17]. Another study that focused on epigenetic alterations triggered by adverse olfactory experiences showed that the unpleasant acetophenone odor was able to regulate the methylation pattern around the *Olfr151* gene, leading to the offspring having an enhanced sensitivity to the unpleasant odor [21]. Indeed, the *Olfr151* gene functions as an odorant receptor in mice, and according to the study, the *Olfr151* locus was observed to be hypomethylated in the sperm of both the F0 and F1 generations. These results also suggest that epigenetic inheritance via DNA methylation occurs in the *Olfr151* locus. The above-mentioned papers show that aberrant methylation profiles in particular loci are correlated with an enriched environment, but there is currently no strong evidence to support the hypothesis that adverse experiences lead to dysregulation of gene expression by altering genome-wide DNA methylation patterns. This is partly due to the lack of genome wide DNA methylation studies on behavioral epigenetics, and the phenotypes of behavioral epigenetic are often difficult to follow (i.e., mental dysregulation). 

### 2.2. Histone Modification

Post-translational histone modification is one of the important chromatin remodeling processes that modulate the chromatin structure around DNA. There are several types of modifications involved in chromatin remodeling, such as acetylation and methylation.

Modification of the chromatin structure leads to an alteration of gene transcription activity. In behavioral epigenetics, most histone acetylation occurs at particular promoters and is correlated with specific gene activity. In an epigenetic research, exposure of mice to chronic social defeat stress model [27] in the form of threat leads to decreased expression levels of *BDNF* (brain-derived neurotrophic factor) genes [18]. In short, downregulation of H3 and H4 acetylation was observed after exposure to chronic social defeat stress.

Histone methylation occurs mainly on the side chains of lysines and arginines and does not alter the charge of the histone protein, unlike acetylation and phosphorylation [28]. H3K9, H3K27, and H4K20 are lysine methylation sites that are associated with transcriptional repression. For instance, methylation at H3K9 is associated with euchromatic gene silencing as well as the formation of silent heterochromatin. Likewise, H3K27 methylation has been implicated in the silencing of *Hox* gene expression. Additionally, H3K27 exhibits a similar mechanism in the silencing of the inactive X chromosome during genomic imprinting. These findings hypothesized that epigenetic alteration localized to the H3K9 gene are likely passed down to the offspring [29]. 

Notably, the enrichment of H3 lysine 27 dimethylation (H3K27) at *BDNF* is observed after mice were treated with the chronic social defeat stress model [18]. It is known that the *BDNF* gene controls the adaptation of the hippocampus to stress and antidepressants. In this case, the histone modifications induce downregulation of *BDNF* mRNA transcription levels, and as a result, the defeated mouse continues to exhibit avoidance behavior. Moreover, the alterations in *BDNF* chromatin structures were retained after a month, which suggests that histone modifications induced by environmental enrichments are long-term adaptive changes and the aberrant histone profiles might remain as an epigenetic mark that plays a role as transcriptional regulators in the hippocampus of mice. In addition, there is evidence from other epigenetic studies suggesting that histone modifications and DNA methylation collaborate with each other to regulate gene transcription activity [30]. For instance, low levels of histone acetylation may induce DNA methylation and result in permanent gene silencing. However, due to limited epigenetic communication research in the area of behavioral epigenetics, further insights into the communication between various epigenetic marks involved in behavioral epigenetics are crucial for understanding the epigenetic regulation induced by various environmental enrichments even though histone modification is being increasingly recognized as an important molecular process in the field of behavioral epigenetics. 

### 2.3. Noncoding RNAs

There is also evidence that noncoding RNAs (ncRNAs), including microRNA (miRNA), PIWI-interacting RNA (piRNA), and transfer RNA (tRNA), play vital roles in the epigenetic regulatory network. Many RNAs can be classified as ncRNAs. Specifically, we are interested in the possible role of miRNA in behavioral epigenetics. Under certain circumstances, miRNA could serve as an epigenetic marker that can rapidly respond to environmental stimuli and contribute to post-fertilization genomic regulation [31]. In a study on the effects of paternal stress on the HPA axis in mice models, the expression of several genes located in the pituitary and adrenal glands of the offspring was altered as a result of parental lifetime exposure to adverse experiences [19]. To investigate the potential role of miRNAs in the mediated changes of HPA axis transcriptional activity, changes in sperm miRNA and its predicted gene targets were examined. The results showed that the content of several sperm miRNAs such as miR-193, miR-204, and miR-29c were significantly increased following paternal stress exposure. Interestingly, it is known that sperm miRNA may affect the mRNA stored in oocytes upon fertilization, and some of these mRNAs can impact offspring development by altering imprinted loci and regulating DNA methyltransferase (DNMT) variants [32]. Other research also suggest that increasing miRNA levels can lead to the offspring’s HPA axis being dysregulated via the changes of miRNA content and mRNA transcriptional patterns [22,23]. Taken together, these researches also implicate that miRNA regulation following parental exposure to adverse experiences may induce the enrichment of specific stress response gene sets through epigenetic regulation and such alteration is heritable. In summary, miRNAs are usually present at relatively low levels in sperm; increased miRNA levels are likely coincidental with adverse parental life experiences; and regulation of miRNAs is often associated with the modulation of mRNA and epigenetic regulation of DNMT variants.

## 3. Behavioral Epigenetic Inheritance Mechanisms

### 3.1. Germline-Mediated Epigenetic Inheritance

**Figure 1:**
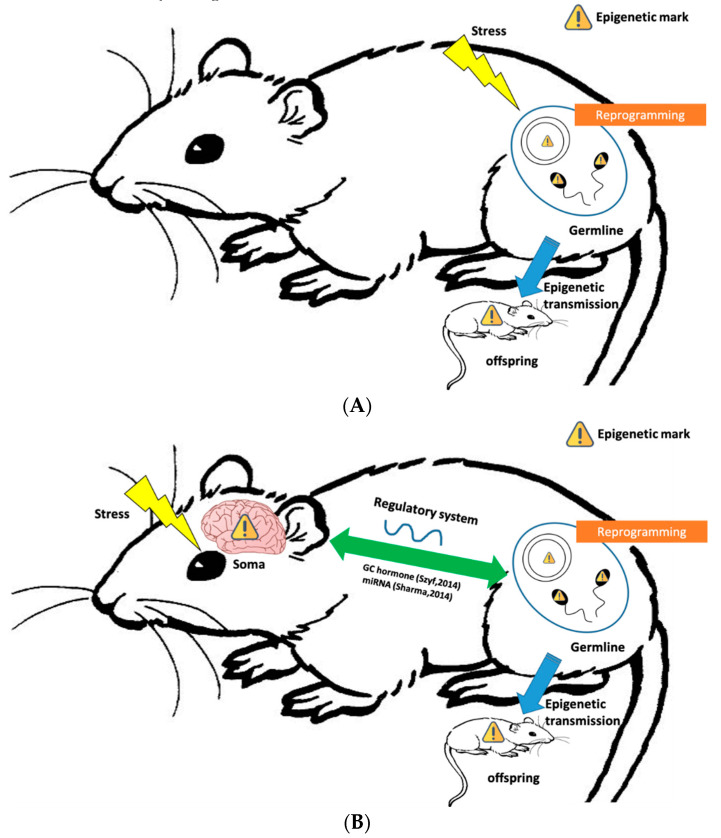
Illustration depicting the potential inheritance pathway in behavioral epigenetics. (**A**) The possible germline-mediated epigenetic inheritance pathway. When mice are exposed to environmental stress, their epigenetic profile may be altered. In addition, reprogramming events occur in the germline, whereby epigenetic markers retained from the reprogramming wave can be inherited by the offspring via germline-mediated epigenetic inheritance. (**B**) An integrated system potentially existing between the soma and germline. In behavioral epigenetics, adverse experiences often trigger alterations of the epigenetic profile in soma; for instance, a lack of maternal care triggers aberrant methylation patterns in genes located among the nervous system. The possible regulatory system existing between the soma and germline suggests that these epigenetic marks are heritable. GC hormones and miRNAs are potential information carriers that circulate epigenetic information between the soma and germline, making it possible for the germline to acquire epigenetic information from soma and pass down the information to the offspring.

The mechanism underlying epigenetic inheritance is currently at the forefront of epigenetic research. For instance, evidence supporting germline-mediated epigenetic inheritance in mammals has increased in the past few years [33]. Notably, the involvement of DNA methylation in germline-mediated epigenetic inheritance is often discussed. It is known that DNA methylation undergoes several resetting processes during the mammalian life cycle, during which other epigenetic markers are reset as well [34]. Figure 1 shows the germline-mediated epigenetic inheritance pathway. In germlines, the DNA methylation profile first experiences global erasure during gametogenesis, which takes place before primordial germ cells differentiate into gametes (sperm and oocytes) [35]. The remethylation program then continues and is completed before birth. The remethylation progress at this stage is asymmetric between female and male germ cells, whereby the sperm completes the cycle first, followed by the oocytes. The second stage of the resetting event occurs during early embryogenesis. The demethylation starts during the preimplantation stage, and during this stage, some of the methylated sites may escape erasure and end up as epigenetic markers. The retention of the DNA methylation profile suggests that epigenetic information is carried over and may be transferred to subsequent generations.

In addition, the CGIs and DNMT variants are often associated with the retention of methylated sites [36,37]. As mentioned above, some behaviors may induce alterations in the DNA methylation profile and these alterations may influence gene expression and even be inherited by the next generation. This finding indicates that, in certain loci, the enriched environment-induced aberrant DNA methylation profile can be transmitted to subsequent generations via the germline. In genes such as *CB1* in mice, the aberrant methylation profile observed following adverse experiences brings about a similar methylation pattern in subsequent generations. This finding thus suggests that epigenetics factors are transgenerational inheritable [8]. However, not all epigenetic marks can be retained during the transmission process, suggesting that a proofreading mechanism exists to regulate DNA methylation. To date, the mechanisms underlying epigenetic proofreading remains under-studied [38]. Furthermore, the DNA methylation state is sensitive to environmental changes; in some scenarios, the transmission line may break when the offspring is exposed to aggressive stimuli during its sensitive exposure period.

Additionally, similar resetting events also occur during histone modifications whereby the nucleosomal histone in sperm are globally erased and replaced by protamine to facilitate DNA packaging activity. The protamine is then removed and new histones are integrated during the fertilization process. In all these resetting events, a fraction of the epigenetic marks may resist protamine replacement, and get inherited by the offspring as a result [39]. Recently, a comprehensive review also suggested that histone modifications could transmit epigenetic information across generations [40]. In particular, certain genes may maintain H3 lysine 27 trimethylation (H3K27me3) via regulation of the polycomb complex, during which the polycomb group proteins are able to retain epigenetic information via enrichment at the promoters of developmental genes. While histone modifications may initiate epigenetic inheritance, there is only an indirect involvement of histone modifications in experience-dependent epigenetic inheritance [41].

### 3.2. Integration between Germline and Somatic Cells in Epigenetic Inheritance

In some ways, epigenetic inheritance may involve cells from germline to soma. The occurrence of epigenetic inheritance due to somatic cells and germline interaction was first demonstrated via examining the multigenerational impact on liver fibrosis after the application of carbon tetrachloride (CCl4) treatment in a rat model [42]. In fact, changes in the DNA methylation status at specific CpG dinucleotides in promoters of antifibrogenic associated genes such as PPAR-γ and TGF-β1 are found to have roles in adaptive responses to liver fibrosis. Research has also found that the adaptive changes that suppress the hepatic wound-healing response can be transmitted to the next generations. This study proposes that there might be an association between liver fibrosis and the germline. This hypothesis is further tested via the regulation of a soluble mediator in CCl4-injured rats, as sperm cells are the only medium transferred from the CCl4-injured rats to their offspring. Furthermore, the soluble mediator in CCl4-injured rats appeared to be consistent with chromatin remodeling in germ cells. Therefore, we make an assumption that epigenetic alterations induced by adverse experiences occurring frequently in somatic cells, such as aberrant DNA methylation profiles caused by enriched environment in the HPA axis, might transfer from the soma to germline via an unknown system. In order to determine the relevance of interact epigenetic inheritance of somatic cells through germline, the connections between the nervous system and spermatogenesis are keys to understanding the integration system in epigenetic inheritance [43].

As mentioned in Section 2.1, parental olfactory experience-induced aberrant *Olfr151* DNA methylation profiles are observed in both the sperm and brain regions [19]. Given the observations regarding sperm acquiring epigenetic information, the circulation of odorants through blood may activate relevant olfactory receptors in sperm. In this study, GC hormones and miRNAs were the targeted mediators of epigenetic information transfer between olfactory tissue and sperm. Other studies have also suggested that extracellular vesicles such as exosomes that consist of polypeptides, DNA, and RNA variants could serve as potential information carriers involved in epigenetic inheritance [44].

In addition, adverse experiences often trigger the alteration of the epigenetic profile in the soma. For instance, adverse maternal care might lead to different miRNA expression levels in young mice [23]. As shown in Figure 1B, the lack of maternal care (stress) might trigger aberrant methylation patterns in genes associated with the nervous system. For these methylation patterns to be inherited, a regulatory system between the soma and germline must be existent. Interestingly, in some cases where the miRNAs are extracted and injected into oocytes, the resulting mice exhibited some alterations in the stress response that differed from the stress response of unaffected mice [19]. Furthermore, the guiding roles of miRNAs in DNA methylation and histone modification [45] also imply that miRNA may be an epigenetic information carrier between somatic cells and the germline. Lastly, although the underlying molecular mechanism between soma and germline is not well understood yet, consistency in observation of epigenetic inheritance across generations will likely suggest an emerging integrative model in behavioral epigenetic inheritance.

## 4. Considerations in the Establishment of Epigenetic Transgenerational Inheritance

*A^vy^* and *Axin^Fu^* are well-known examples of metastable epialleles in mammals and epigenetically regulated phenotypes such as agouti coat color and kinked tail are observed to be transmitted across generations [46,47]. While there are stably inherited epialleles found in both animals and plants, there remains much uncertainty regarding transgenerational inheritance in behavioral epigenetics.

First, a precise understanding of the definition of epigenetic transgenerational inheritance is essential. Epigenetic transgenerational inheritance is generally described as the transmission of nongenetic information across at least two generations in the absence of direct environmental exposure [48]. For transgenerational inheritance, patrilineal and matrilineal lines are considered to be different; in some previous research, epigenetic transgenerational inheritance was misidentified as an effect of multigenerational exposure. For instance, when the gestating female (F0) was exposed to environmental stress, the primordial germ cell (F1 germ cell) carrying the gestating female was considered to be exposed as well. Thus, examination of transgenerational inheritance effects in matrilineal lines should start from the F3 generation. In contrast, assessment of transgenerational inheritance in patrilineal lines can start from the F2 generation because there is no exposure in the gestating period during patrilineal transmission. Furthermore, even though postnatal experience is also an important concern in experience-dependent epigenetics, there are many interfering factors, such as in utero effects that should be taken into consideration when examining the postnatal effects [49,50]. Thus, to address the difficulties associated with behavioral epigenetic research, a well-designed experiment is especially important. For instance, cross-fostering is crucial in determining the contribution of postnatal experience in epigenetic inheritance [51]. In short, an understanding of the fundamental concept of epigenetic inheritance and precise experimental design are necessary to study epigenetic transgenerational inheritance mechanisms in behavioral epigenetics.

Second, an understanding of how epigenetic profiles can be inherited meiotically and sustained in subsequent generations is required. As mentioned above, the epigenetic profile in the genome is subjected to two dynamic reprogramming events during the formation of germ cells and before embryo implantation [52]. Most biologists have linked epigenetic transgenerational inheritance to the incomplete erasure of epigenetic markers during the occurrence of reprogramming events before embryo implantation in the uterus. To address this issue, scientists have focused on the differentially methylated region (DMR) and differentially methylated gene (DMG) that often escape demethylation during reprogramming waves and hence get expressed in subsequent generations. Recently, one comprehensive review suggested that the escape of methylated regions from reprogramming waves is not a stochastic event, and DNMT variants have exhibited significant roles in the regulation of the methylation pattern [53]. Hence, an underlying pattern of epigenetic regulation may be observed via the examination of epigenetic profiles across a variety of species. For instance, the methylation profile in humans in the previously mentioned CGIs is usually retained after the reprogramming event. Therefore, CGIs are selectively subjected to imprinting.

Another consideration in epigenetic transgenerational inheritance is the transmission of epigenetic information in only matrilineal or patrilineal lines, indicating a sex-specific epigenetic inheritance model [54,55]. The sex-specific inheritance model indicates a complicated regulatory network underlying the incomplete erasure of epigenetic markers on either parental side. A previous review proposed that the specialized chromatin structure is required to protect CGIs from demethylation [56]. Furthermore, clustering of imprinted genes is associated with the existence of imprinting control regions, whereby clusters of methylated imprinted genes can have a higher probability of survival across reprogramming events. In this scenario, both paternal and maternal effects in behavioral epigenetic inheritance should be considered, as most research in the field of behavioral epigenetics is associated with only the exposure of adverse experiences during the gestation period. Perhaps the specific escape pattern during epigenetic reprogramming events in behavioral epigenetic cases will be revealed in future research, as the complex mechanisms underlying these events are substantial obstacles for the establishment of epigenetic inheritance mechanisms in mammals.

Recently, epigenetic marks associated with environmental enrichment have been predicted to be reversible by several studies. One of the studies suggests that treatment with the histone deacetylase (HDAC) inhibitor trichostatin A (TSA) can reverse the hypermethylation status induced by maternal behavior in the exon 1_7_ GR promoter [57]. According to mapping results, TSA treatment decreased the methylation level of the maternal care group by increasing the K9 acetylation of H3 histones in the exon 1_7_ GR promoter. In short, the DNA methylation profile in the hippocampus can be reversed through exposure to other aggressive stimuli that can alter chromatin structure. Therefore, in order to be transgenerationally inherited, the epigenetic markers should at least be sustained before conception.

Furthermore, accumulating evidence supports a possible role for in vitro fertilization (IVF) treatments in alternating the stability of epigenetic markers such as DNA methylation and histone modifications [58]. In a mouse study, in vitro embryo cultures exhibited decreased growth rates and organ mass of offspring. Similar phenomena were observed in the subsequent generation as well [59]. As indicated above, IVF treatments can disrupt epigenetic regulation. Therefore, it is crucial to understand the implications of IVF in behavioral epigenetics as some of the offspring in behavioral epigenetics research is conceived via IVF.

The interact epigenetic inheritance of somatic cells through germline also led to the consideration of the epigenomic impact of epigenetic information carrier circulated between somatic cells and the germline during the inheritance process. However, there is more than one regulatory factor that is suggested to be involved in the circulation of epigenetic information. To be transgenerationally inherited, interplay between epigenetic markers is especially important. Several studies have suggested that epigenetic markers that are transmissible to subsequent generations will be affected if one of these markers is absent or loses its function due to interference. For instance, regulation of DNMT variants is often linked to histone modifications in the induction of gene silencing [41]. Without a clear understanding of the epigenetic inheritance mechanism, it is difficult to firmly state that epigenetic material can be transmitted via only one epigenetic mechanism. Further research is thus needed to study the cooperation between epigenetic markers.

Lastly, genetic effects should not be ignored in epigenetic studies. A study on the contribution of genetic variation to epigenetic inheritance in humans suggested that up to 20% of variation in DNA methylation profiles is due to the sequence-based DNA variants [60]. Thus, in addition to environmental enrichment as the source of stimuli in behavioral epigenetic regulation, genetic factors may play a particularly important role in epigenetic inheritance.

## 5. Conclusions

Behavioral epigenetics is an interdisciplinary field involving neurobiology, psychology, reproductive biology, etc. Research in behavioral epigenetics over the past few years has revealed that adverse experiences associated with environmental enrichment are able to induce alteration in epigenetic marks. Hence, further investigation is required to answer important questions concerning epigenetic regulation upon exposure to an enriched environment, and the impact of such epigenetic regulation. Epigenetic alterations such as DNA methylation and histone modification are among the most discussed mechanisms mediated by adverse environmental enrichment, which includes childhood abuse, maternal separation, chronic stress, etc. Moreover, the regulation of noncoding RNAs, such as miRNA, has emerged as a potential mediator of epigenetic effects that may change the transcriptional activity of genes associated with the nervous system after adverse stress exposure. In some cases, the epigenetic marks mentioned might interact with each other to regulate the transcriptional activity. For instance, low levels of histone acetylation may induce DNA methylation, which are likely to result in permanent gene silencing. However, despite our current understandings on behavioral epigenetics, epigenetic communication research merits further investigation.

Furthermore, germline-mediated epigenetic inheritance, subsequent effects on somatic cells, and the germline inheritance model are proposed and explained in the review. For instance, our review concludes that epigenetic inheritance models provide a potential pathway for these epigenetic marks to be passed down to the offspring and that there is a possible restriction of epigenetic inheritance due to reprogramming events. In addition, current observations of behavioral epigenetics are specific to certain stress response loci, making it unclear whether there is a direct impact of enriched environment on genome-wide epigenetic regulation. Overall, researchers are far from deciphering the model of epigenetic inheritance because the current scope of epigenetic research is not comprehensive enough.

Another promising area of behavioral epigenetic is the establishment of transgenerational epigenetic inheritance. Epigenetic transgenerational inheritance in behavior occurs when similar behaviors shown in both parents and offspring sustain two generations and beyond in absence of direct environmental exposure. However, there are some limitations in conducting such interdisciplinary research. For instance, standardized guidelines for experimental design is needed to determine the mechanism underlying epigenetic inheritance, as most of the inducing stimuli lack consistency, leading to incoherent results. In addition, sex-specific epigenetic inheritance models and the potential reversibility of epigenetic information need further investigation to narrow or bridge the gap in our understanding of epigenetic transgenerational inheritance. Understanding the communication between epigenetic marks and genetic effects is also crucial as most of epigenetic alterations occur differently depending on the DNA sequences. In conclusion, further work such as long-term experiments and the development of advanced technologies is required to understand this topic. Hopefully in-depth research can lead to breakthroughs in the epigenetic field.

## Figures and Tables

**Table 1:** Experience-dependent epigenetics inheritance studies.

Species	Inducing Conditions	Acquired Traits	Locus	Transmission Stability	Epigenetic Marks	Profiling Methods	References
Human	Childhood abuse (sexual contact, severe physical abuse, negligence)	Decreased expression of the glucocorticoid receptor 1F variant	*NR3C1* promoter 1F	F1	DNA methylation	Sodium bisulfite sequencing	McGowan et al., 2009 [7]
Mouse	Chronic stress (immobilization)	Alteration HPA axis that trigger long-term stress response	*NR3C1* promoter 17	F1	DNA methylation	Pyrosequencing	Witzmann et al., 2012 [17]
Mouse	Unpredictable maternal separation	Depressive-like behavior	*MeCP2*, *CB1*, *CRFR2*	F1-F3	DNA methylation	Pyrosequencing	Franklin et al., 2010 [8]
Mouse	Chronic social defeat stress–social avoidance	Depressive-like behavior: less interactive in the population	*BDNF* gene	F1	Post translation histone modifications	ChIP assays	Tsankova et al., 2006 [18]
Mouse	Paternal chronic stress exposure	Increased miRNA variants expression: HPA stress axis dysregulation	CRFr1, POMC, Mc2r, 11βHSD-1	F1	miRNAs	TaqMan Array microRNA	Rodgers et al., 2013 [19]
Rat	Adverse maternal care	Aberrant BDNF DNA methylation result in atypical adverse maternal behavior	*BDNF* gene	F1-F2	DNA methylation	MeDIP-seq	Roth et al., 2009 [20]
Mouse	Parental olfactory experience	Hypomethylation: Increased behavioral sensitivity to acetophenone (odor)	*Olfr151* gene	F1-F2	DNA methylation	Illumina sequencer	Dias & Ressler, 2014 [21]
Mouse	Prenatal stress exposure	Dysmasculinization		F1-F2	miRNAs		Morgan & Bale, 2011 [22]
Mouse	MSUS (unpredictable maternal separation & stress)	Altered metabolic response		F1-F3	miRNAs & piRNAs		Gapp et al., 2014 [23]
Rat	Prenatal chronic restraint stress	Dysregulation of offspring gene expression and impact on offspring neurodevelopment system	*HSD11B2*	F1	DNA methylation	Pyrosequencing	Peña et al.,2012 [24]

## Abbreviations

HPA	Hypothalamic-pituitary-adrenal
